# Immunization Coverage of Inmates in Spanish Prisons

**DOI:** 10.3390/ijerph17218045

**Published:** 2020-10-31

**Authors:** Nancy Vicente-Alcalde, Jose Tuells, Cecilia M. Egoavil, Esther Ruescas-Escolano, Cesare Altavilla, Pablo Caballero

**Affiliations:** 1 Penitentiary Center Alicante II, General Secretariat of Penitentiary Institutions, 03400 Villena, Spain; nanvical@gmail.com; 2 Department of Community Nursing, Preventive Medicine, Public Health and History of Science, University of Alicante, 03690 Alicante, Spain; eatingfaster@gmail.com (C.A.); pablo.caballero@ua.es (P.C.); 3 General University Hospital of Alicante, Clinical Pharmacology Unit, 03690 Alicante, Spain; egoavil_cec@gva.es; 4 Servicio de Urgencias, Hospital Universitario del Vinalopó-Elche, 03293 Elche, Spain; esther.ruescas@gmail.com

**Keywords:** immunization coverage, prisoners, hepatitis A, hepatitis B, tetanus, diphtheria pneumococcus, seasonal influenza

## Abstract

The correct immunization of the inmate population minimizes the risk of transmission of vaccine-preventable diseases in prisons. The objective of this study was to evaluate the vaccine coverage of long-term prisoners in the Spanish penitentiary system through a retrospective longitudinal study. One-thousand and five prisoners were selected, who were imprisoned from 2008 and 2018 in three Spanish prisons. Their degree of immunization was evaluated as related to hepatitis A (HAV), hepatitis B (HBV), tetanus, diphtheria, pneumococcus and seasonal flu. The state of vaccination of the prisoners with a serological diagnosis of HBV, hepatitis C (HCV) and human immunodeficiency virus (HIV) was also evaluated. The vaccination coverage obtained for hepatitis B was 52.3%, and for tetanus–diphtheria, it was 71.9%. However, for hepatitis A and pneumococcus infection, it was insignificant (<2% of the prisoners). Vaccination against seasonal flu was lower than 16%. The HCV and HIV-positive inmates were not correctly vaccinated either. The insufficient level of immunization obtained reflects the lack of interest and marginalization of this population by the penitentiary system and the health authorities. The lack of reliable records is combined with the lack of planned strategies that promote stable and well-defined programs of active vaccination.

## 1. Introduction

The undoubtable benefit of vaccination as a measure of public health in terms of efficacy and cost-effectiveness is of particular interest in the prison environment. The inmate population has characteristics that increase its vulnerability for suffering transmissible diseases, and some of them are preventable through immunization [1]. For this population, the passage through prison is an excellent opportunity for coming into contact with the health system, as it is a population that is difficult to access in the community, and a large number of prisoners are part of marginalized groups or disadvantaged sectors of society [1]. The importance of vaccination in prisons is a benefit for society, as their contact with the exterior is frequent [2]. Being a closed community facilitates the implementation of immunization strategies by the health professionals of the penitentiary institutions [2]. The infectious disease that has been the greatest focus of these interventions has been HBV, given its mechanism of transmission, with a high risk among parenteral drug users [3,4,5,6,7,8]. A recent review conducted to show existing evidence about vaccines in penitentiary centers in the EU/EEA included studies about vaccines against HBV, HVA, Influenza and Triple vaccine (MMR). The United Kingdom is the country where most of the studies on vaccination coverage in prisoners were published, followed by the United States, and France, and to a lesser extent, Spain, Italy, Luxembourg, Canada, Switzerland and Denmark [9].

The twelfth worldwide report informed that the worldwide prison population was over 11 million. At present, Spain has a total of 58,517 inmates (92.53% men and 7.45% women) [10], with a ratio of 125 inmates per 100,000 inhabitants, occupying an intermediate place with respect to the rest of the world according to The World Prison Brief (PrisonStudies.org) and much lower than those that exceed 500 × 100,000 (USA, El Salvador, Turkmenistan, Thailand, Cuba), although the Spanish Penitentiary System is the second-largest in Southern Europe after Italy [11].

In October 1989, vaccinations begun in Spanish prisons in a systematic manner, following the schedule from the Ministry of Health, which in a recent document, recommended vaccination against HBV (three doses), and an update of the MMR, aside from offering the vaccine against the flu [12]. The objective of this study was to evaluate the vaccination coverages of HAV, HBV, tetanus–diphtheria, pneumococcal disease and seasonal influenza, among prisoners of the Spanish prison system.

## 2. Materials and Methods

### 2.1. Study Design

**Figure 1:**
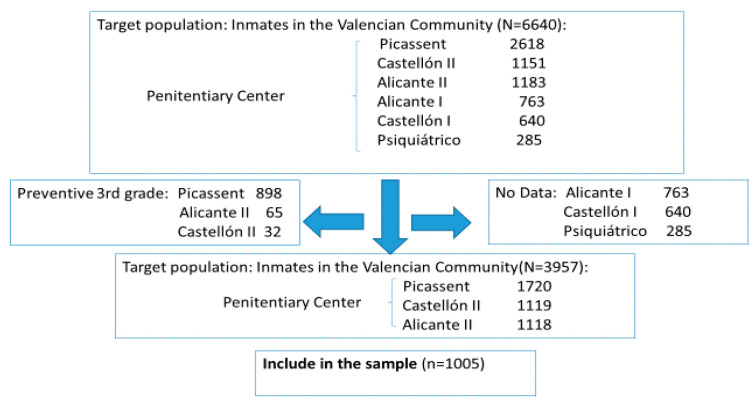
Sample selection.

A retrospective, longitudinal study was conducted on vaccine coverage of inmates imprisoned in Spanish prisons during the period ranging from 1 January 2008, to 31 December 2018, with data provided by the computer programs SANIT and OMI from the Spanish Penitentiary Administration. The study population was selected through simple randomized sampling and included those who complied with the following requisites: convicted inmates, non-preventive or under an open regime, older than 18, male or female, interned in three penitentiary centers (Penitentiary Center Alicante II, Penitentiary Center Picassent, and Penitentiary Center Castellon II). The following centers were excluded: Psychiatric Penitentiary Center of Foncalent, the Penitentiary Center Alicante I, and the Penitentiary Center Castellon I, because during part of the 2008–2018 period, these centers did not comply with the requisite of having computerized records of vaccination or serological tests in the SANIT and OMI program. The sample size was determined to estimate the ratios of vaccination on a population of 6640 interns (penitentiary population of the Valencian Community as of December, 2018), with a confidence interval of 95%, and error of estimation of ±3% and a most unfavorable proportion of 0.50, which resulted in sample size of 925 subjects. This was increased by adding a quota of 10% due to possible losses, resulting in final size of 1005 subjects (Figure 1).

### 2.2. Data Collection

The collection of data was conducted by nursing personnel affiliated to the Penitentiary Institutions Nurse Corps from each center studied, after the previous authorization from the Sub-Directorate General of Institution Relations and Territorial Coordination of the Ministry of Interior. The electronic records were examined in alphabetical order. The following variables were recorded: age, sex, and nationality of the prisoners, and penitentiary center. The state of immunization was recorded with the number of doses administered in prison or prior, of the vaccines against HAV, HBV, tetanus–diphtheria, pneumococcus disease, and seasonal flu. Serological data of infection was also collected for HBV, HCV and HIV.

The vaccination schedule recommended for the adult vaccination calendar from the Ministry of Health was consulted. Inmates who were correctly immunized were those who had received 2 doses of the vaccine against hepatitis A (HVAv), 3 doses of the vaccine against hepatitis B (HBVv), 3 doses of the vaccine against infection by tetanus–diphtheria (Tdv), 1 dose against Pneumococcal disease (PPv23), and 1 dose against the seasonal flu per year. The inmates who had not received any vaccines, as well as those who did not have any records in their electronic data were considered to be non-immunized. The inmates who had received less doses than those required were considered non-immunized as well. The inmates who had received more doses than those required by the vaccination schedule were considered to be vaccinated. The commercial names of the vaccines utilized during the period analyzed were not provided because these were not included in the records.

For the inmates with HIV and HCV, the same schedules and doses needed were considered for classifying them as correctly immunized. Moreover, the doses of the vaccines received by the inmates prior to their imprisonment was also recorded (previously immunized).

### 2.3. Statistical Analysis

To describe the characteristics of the study population descriptive statistics were used. The means and standard deviations were calculated for quantitative variables, chi-square test and Fisher’s exact test (categorical variables) were used to compare variables and to evaluate association between vaccination coverage for HAV and HBV.

To assess the association between the independent variables and the two populations, odds ratios (OR) were estimated with the “odds ratios” (OR) statistic and the corresponding confidence intervals at 95%. Differences were considered statistically significant when *p*-values < 0.05. Statistical evaluations were performed using SPSS v20 software (SPSS Inc., Chicago, IL, USA).

### 2.4. Research Ethics

The study was approved by the General Secretary of Penitentiary Institutions, Sub-directorate General of Institutional Relations and Territorial Coordination, (08/03/2018 number 395714). The information was treated confidentially, and in accordance with Organic Law 3/2018, from December 5th, on Protection of Personal Data.

## 3. Results

### 3.1. Participants’ Characteristics

The final sample of 1005 participants were comprised of a total of 937 (93.2%) men and 68 (6.7%) women (Table 1). The mean age of the inmates was 38 years old. Most of the prisoners (71.6%) were found in the 30 to 60 age range, and only 4.6% exceeded the age of 60. There were 866 (86.2%) Spanish prisoners and 139 (13.8%) prisoners from other nationalities. Of these, 29.5% were Moroccans, 19.42% were Romanian, 11.5% were Colombian, 7.9% were Algerian and the rest of the nationalities were below 5% (Table 1).

### 3.2. Vaccination Coverages

Most of the prisoners (98.2%) were not immunized against HAV infection. However, those vaccinated against HBV only reached a total of 52.3%, of which 75.7% had completed the correct schedules. Considering the variables sex and country of origin, great differences were not found between men or women or between Spanish or foreign nationals. Although the inmates older than 60 were the most vaccinated (55.3%), only 61.5% had completed the vaccination schedule. Among those who were aged between 30 and 60, this complete schedule ascended to 79.4%, thus becoming the best-vaccinated age group (Table 2).

The vaccination against tetanus–diphtheria (Tdv) reached a total of 71.9%, of which 58.4% had received the complete schedule. The women received the greatest number of Tdv doses (82.4%). However, more men had complete vaccination schedules (59.2%). Starting at 30 years of age, a higher percentage of vaccination was observed (74.5%) for Tdv. The Spanish citizens had received the greatest number of doses (72.4%), but the foreigners had the most complete vaccination schedules (60.4%). Against Pneumococcal disease, 99.2% of the inmates were not immunized (Table 3).

During the study period (2008–2018), the vaccine against seasonal flu was offered to the inmates. An increase in the vaccination rate was observed in the 2010–2013 period after the 2009 H1N1 Pandemic (H1N1pdm09 virus), reaching values of 16.2% vaccinated inmates. This percentage decreased during the posterior vaccination campaigns (2014–2018), a period in which the electronic recording of data decreased, as the vaccinations against the seasonal flu were not recorded correctly.

The OR calculation could only be applied to the vaccines against HBV and Td, as the data were insufficient for the rest of the vaccines. An association between the variables sex and country of origin was not found for vaccination rates. Age had a protective association in the inmates aged 30 to 60 for HBV (OR: 1.6; 95% CI: 1.2–2.2) and Td (OR: 1.8; 95% CI: 1.3–2.4), as well as for the group older than 60 for the Td vaccine (OR: 2.1; 95% CI: 1.1–4.1) (Table 4).

### 3.3. Vaccination Coverages in Prisoners with Positive Serology of HCV, HBV and HIV

Serological tests had been performed for the inmates against HIV (81.2%), HBV (surface antigen of the hepatitis B virus (HBsAg), 78.9%) and HCV (78.4%). Of the inmates, 7.8% were infected with HIV and 20.5% with HCV. In the case of HBV, 19.7% had been previously infected with the disease, and 3.8% had an active infection (Table 5).

The prisoners infected with HIV entered the prison with a rate of previous immunizations against HBV of 26.5%, and against Td of 39.1%, however, this vaccination coverage did not improve during their stay in prison. In the case of those who were HCV positive, a similar phenomenon was observed. Although they had entered prison with a previous immunization rate of 22.4% against HBV, and 33.5% against Td, the coverage did not increase during their stay in prison. The vaccination rate against HAV and pneumococcal disease was inexistent (Table 6).

As for HBV, it was observed that for an acute infection (HBsAg-positive), 60% of the inmates did not have a record of vaccines doses, and 40% were over-vaccinated. Moreover, 41.2% of the inmates with a HBsAb-positive serological result (immunized through vaccines) did not have a record of vaccination dose, and 7.3% had exceeded the recommended dosage. Lastly, 72% of the inmates who had had the disease (HBcAb-positive), did not have a vaccination record, and 28% had received vaccines after overcoming the disease (Table 7).

## 4. Discussion

To our knowledge, this is the first study that has analyzed the coverage of various vaccines in prisons. Generally, studies are centered on vaccines against HBV [4,5,6,7,8,13,14,15], HAV [16,17,18,19], and to a lesser degree, to seasonal flu [20,21] and Pneumococcal disease [22,23,24].

Our results show a vaccine coverage against HBV (52.3%) higher than that obtained in studies conducted to improve vaccination strategies, which showed coverage values between 25% and 40% [8,25,26,27], although it is inferior to other studies with coverage rates ranging from 63% to 82.6% [5,6,7].

Among the reasons for non-vaccination or incomplete vaccination, the following was pointed out: not offering the vaccines to the inmates, that they did not follow through with the posterior doses either due to not wanting it, release from prison or transfer, or not wanting to be vaccinated due to fears of the secondary effects [28,29].

The vaccinations performed in other countries against HAV show coverage rates between 60% and 96% [17,18,30]. The almost null vaccination rate in our study shows the lack of interest of the penitentiary and/or health authorities for implementing this vaccine. Furthermore, the prevalence of HBV and HVC in prison requires improvements in the vaccination strategy against HAV, as it increases the risk of liver complications [31,32].

Various studies have shown that it is possible and effective to implement a program of immunization among the inmates, at least for HBV and HAV [33,34], considering the potential losses in health and disability [35,36].

Somewhat more than half (58.4%) of the inmates were correctly vaccinated against tetanus and diphtheria, insufficient coverage for a vaccine that should be routine, also considering the relationship between the parenteral drug users and the appearance of tetanus cases [37,38]. The review by Madeddu et al. confirms the inexistence of studies on vaccine coverage against Td of inmates [9].

The flu has special characteristics as compared other vaccine-preventable diseases [39]. According to the WHO, influenza continues to be one of the greatest threats to public health and recommends vaccination for patients who have hepatitis and HIV [40], and this is where the importance of vaccination in penitentiary centers lies, which is still deficient according to our study. This is similar to what is observed with the administration of the vaccine against pneumococcal disease, recommended for institutionalized individuals and those older than 60 years old [41,42,43]. However, a clear strategy is lacking for its implementation in penitentiary institutions, the international context or in our study. Immunization records were not found for chickenpox or MMR, as this is not recommended for inmates in Spain, although paradoxically, it is recommended for penitentiary system workers [12].

The serological analysis in our study showed very complete data for infections of HBV, HCV and HIV, which exceeded the data obtained in other studies [44,45,46]. The prevalence of HIV and HCV is greater for inmates than the general population [47]. In addition, they are over-exposed to other hepatitis, respiratory illnesses and influenza [48,49,50], so that the low percentages obtained for HBV vaccination (<5%) and Td (<9%) and the almost non-existent percentages for HAV and pneumococcal disease are discouraging.

The frequency of transfers between the different penitentiary centers in Spain allows the attainment of a good general perspective of the immunization coverage in the penitentiary system in Spain. The most important result found was to verify the low level of vaccine coverage in the last decade in Spanish prisons. This is the first study that offers data on the last ten years on immunization of preventable diseases such as HBV, HAV, tetanus/diphtheria, pneumococcal disease and influenza in Spanish prisons, which, when combined with positive serological data of the prisoners for HBV, HBC and HIV, contributes results with an additional clinical relevance.

As for the limitations of the study, we can point out that the data collected corresponded to long-term prison inmates, who have had a greater opportunity for receiving vaccines, as compared to inmates who have short sentences or preventive prisoners for whom exclusion criteria were applied. These could have worst coverage results as observed in a previous study [8]. Moreover, three penitentiary centers had to be excluded due to the lack of an official computerized records system during part of the study period. The overestimation or underestimation of coverage has not been questioned, the results obtained in the study are far from being desirable, and are not optimal according to those recommended for the general population in vaccination programs.

In the last 30 years, the preventive strategies have contributed to limit the propagation of hepatitis B [51,52], a disease that is associated to the consumption of drugs in prisons [18,28,37,45]. Increasing immunization coverage should be combined with improvements in the drug policies in the prison environment.

Promoting vaccination could avoid health problems and hospitalizations, minimizing the additional economic and organizational load for the penitentiary system. The inmates affected by transmissible diseases act as propagators within the prison, either to other inmates, as well as penitentiary workers and the community itself [38], and there is a high load of morbimortality that is preventable if immunization programs are applied.

## 5. Conclusions

In Spain, there are notable shortcomings in the application of vaccination policies in penitentiary centers. There is a lack of a good coordination between the penitentiary system and the National Health Service. The latter provides the recommendations about the necessary vaccines in the area of prisons, which are different if dealing with a prisoner or a worker. Furthermore, it freely provides and distributes the vaccines doses for their administration to the prisoners but does not demand data about which and to whom they have been applied.

The penitentiary system does not apply vaccines to preventive prisoners or those without a firm sentence, which signifies an example of the loss of opportunity for vaccination, and a prisoner can spend two years as a preventive prisoner without receiving a single vaccine dose. The most severe problem is the insufficient information on the vaccines administered due to the low quality of the records, poor and incomplete digitalization that impedes knowledge of the true immunization coverage. The current penitentiary records system of vaccines is also not linked to the National Health Service, which marginalizes the penitentiary institution population, which is already vulnerable.

The study has brought to light the insufficient vaccine coverage in Spanish prisons, providing evidence on a poor public health strategy, which could be improved with the correct management. It is not a difficult task that requires a great amount of resources, it is a matter of political will and decision making which presupposes the improvement of vaccine training of health professionals of the penitentiary system, providing them with a tool that works, a data recording system coordinated with the National Health System, providing them with precise instructions about the recommended vaccines and encouraging a culture of immunization between the inmate population with proactive interventions of awareness.

## Figures and Tables

**Table 1:** Distribution of the sample according to study variables.

Total	Characteristics	1005 (*n*)	%
**Penitentiary Center**	Alicante II	509	50.6
	Picassent	199	19.8
	Castellón II	297	29.6
**Country**	Spain	866	86.2
	Not Spain	139	13.8
**Age**	<30 years	237	23.6
	30–60 years	720	71.6
	>60 years	47	4.7
**Sex**	Men	937	93.2
	Women	68	6.8

**Table 2:** Vaccination coverage for HAV and HBV.

Characteristics			HAV				HBV			
			Non Vaccinated	Vaccinated	Complete Schedule	Incomplete Schedule	Non Vaccinated	Vaccinated	Complete Schedule	Incomplete Schedule
		1005	987	18	2	16	479	526	398	128
		100%	98.2%	1.8%	11.1%	88.9%	47.7%	52.3%	75.7%	24.3%
**SEX**	**Men**	937	920	17	1	16	447	490	371	119
		93.2%	98.2%	1.8%	5.9%	94.1%	47.7%	52.3%	75.7%	24.3%
	**Women**	68	67	1	1	0	32	36	27	9
		6.7%	98.5%	1.5%	100.0%	0.0%	47.1%	52.9%	75.0%	25.0%
**AGE**	**<30 years**	237	235	2	0	2	125	112	74	38
		23.6%	99.2%	0.8%	0.0%	100.0%	52.7%	47.3%	66.1%	33.9%
	**30–60 years**	720	705	15	1	14	332	388	308	80
		71.6%	97.9%	2.1%	6.7%	93.3%	46.1%	53.9%	79.4%	20.6%
	**>60 years**	47	46	1	1	0	21	26	16	10
		4.6%	97.9%	2.1%	100.0%	0.0%	44.7%	55.3%	61.5%	28.5%
**COUNTRY OF ORIGIN**	**Spain**	866	850	16	2	14	414	452	342	110
		86.2%	98.2%	1.8%	12.5%	87.5%	47.8%	52.2%	75.7%	24.3%
	**Not Spain**	139	137	2	0	2	65	74	56	18
		13.8%	98.6%	1.4%	0.0%	100.0%	46.8%	53.2%	75.7%	24.3%

Notes: HAV: hepatitis A, HBV: Hepatitis B.

**Table 3:** Vaccination coverage against tetanus–diphtheria and Pneumococcal disease.

			Tetanus–Diphtheria				Pneumococcal Disease	
Characteristics			Non Vaccinated	Vaccinated	Complete Schedule	Incomplete Schedule	Non Vaccinated	Vaccinated/Complete Schedule
		1005	282	723	422	301	997	8
		100%	28.1%	71.9%	58.4%	41.6%	99.2%	0.8%
**SEX**	**Men**	937	270	667	395	272	930	7
		93.2%	28.8%	71.2%	59.2%	40.8%	99.3%	0.7%
	**Women**	68	12	56	27	29	67	1
		6.8%	17.6%	82.4%	48.2%	51.8%	98.5%	1.5%
**AGE**	**<30 years**	237	87	150	81	69	237	0
		23.6%	36.7%	63.3%	54.0%	46.0%	100.0%	0.0%
	**30–60 years**	720	183	537	320	217	713	7
		71.6%	25.4%	74.5%	59.6%	40.4%	99%	1%
	**>60 years**	47	12	35	21	14	47	0
		4.6%	25.5%	74.5%	60.0%	40.0%	100.0%	0.0%
**COUNTRY OF ORIGIN**	**Spain**	866	239	627	364	263	858	8
		86.2%	27.6%	72.4%	58.1%	41.9%	99.1%	0.9%
	**Not Spain**	139	43	96	58	38	139	0
		13.8%	30.9%	69.1%	60.4%	39.6%	100.0%	0.0%

**Table 4:** Statistical associations for HBV and Td (odds ratio (OR)).

		HBV				Td			
Characteristics		Complete Schedule	Non Vaccinated Incomplete Schedule	OR	95%CI	Complete Schedule	Non Vaccinated Incomplete Schedule	OR	95%CI
**SEX**	**Men**	371	566	Ref.		395	542	Ref.	
	**Women**	27	40	1.0	(0.6; 1.7)	27	40	0.9	(0.6; 1.5)
**AGE**	**<30 years**	74	163	Ref.		77	160	Ref.	
	**30–60 years**	308	417	1.6 *	(1.2; 2.2)	324	401	1.8 *	(1.3; 2.4)
	**>60 years**	16	27	1.3	(0.7; 2.6)	21	22	2.1 *	(1.1; 4.1)
**COUNTRY OF ORIGIN**	**Spain**	342	524	Ref.		364	502	Ref.	
	**Not Spain**	56	83	1.0	(0.7; 1.5)	58	81	1.0	(0.7; 1.4)

* Statistical significance < 0.05. HAV and Pneumococcal records were insufficient for the calculation of OR. Notes: HBV: hepatitis B, Td: Tetanus-diphtheria, OR: odds ratio.

**Table 5:** Overall results of serological analysis among inmates (*n* = 1005).

Serology	HIV	HCV	HBV		
			HBsAg	HBsAb	HBcAb
	*n* = 817	*n* = 788	*n* = 793	*n* = 729	*n* = 634
**Positive**	64 (7.8%)	161 (20.5%)	30 (3.8%)	706 (96.8%)	125 (19.7%)
**Negative**	753 (92.2%)	627 (79.5%)	763 (96.2%)	23 (3.2%)	509 (80.3%)

Notes: HIV: human immunodeficiency virus, HCV: hepatitis C virus; HBV: hepatitis B virus, HBsAg: surface antigen of the hepatitis B virus (acute infection), HBsAb: hepatitis B surface antibody (immunized through vaccine), HBcAb: hepatitis B core antibody (past infection). *n* = number of inmates with electronic data.

**Table 6:** Immunization coverage of HIV-positive and HCV-positive inmates.

**HIV-Positive Inmates (*n* = 64)**	**Non-Immunized (%)**	**Immunized (%)**	**Previously Immunized (%)**
HAVv	64 (100%)	--	--
HBVv	46 (71.9%)	1 (1.6%)	17 (26.5%)
Tdv	36 (56.3%)	3 (4.6%)	25 (39.1%)
PPv23	64 (100%)	--	--
**HCV-Positive Inmates (*n* = 161)**			
HAVv	161 (100%)	--	--
HBVv	107 (72.6%)	8 (5%)	36 (22.4%)
Tdv	93 (57.8%)	14 (8.7%)	54 (33.5%)
PPv23	161 (100%)	--	--

Notes: HIV: human immunodeficiency virus, HCV: hepatitis C virus, HAVv: hepatitis A vaccine, HBVv: hepatitis B vaccine, Tdv: tetanus-diphtheria vaccine, PPv23: Pneumococcal vaccine.

**Table 7:** Over-vaccination for hepatitis B of HBsAg-positive, HBsAb-positive and HBcAb-positive inmates.

Doses	HBsAg+ (*n* = 30)	HBsAb+ (*n* = 706)	HBcAb+ (*n* = 125)
Without recorded doses	18 (60%)	291 (41.2%)	90 (72.0%)
Dose 1	4 (13.3%)	51(7.2%)	6 (4.8%)
Dose 2	7 (23.3%)	45 (6.4%)	4 (3.2%)
Dose 3	1 (3.3%)	268 (38.0%)	23 (18.4%)
Dose 4	--	43 (6.1%)	2 (1.6%)
Dose 5	--	7 (1.0%)	--
Dose 6	--	1 (0.1%)	--

Notes: HBsAg^+^: surface antigen of the hepatitis B virus (acute infection), HBsAb^+^: hepatitis B surface antibody (immunized through vaccine), HBcAb^+^: hepatitis B core antibody (past infection). *n* = number of inmates with electronic data.
